# Interpretable machine learning predicts cardiac resynchronization therapy responses from personalized biochemical and biomechanical features

**DOI:** 10.1186/s12911-022-02015-0

**Published:** 2022-10-31

**Authors:** Anamul Haque, Doug Stubbs, Nina C. Hubig, Francis G. Spinale, William J. Richardson

**Affiliations:** 1 Biomedical Data Science & Informatics Program, Clemson University, Clemson, SC USA; 2 School of Medicine, Columbia Veterans Affairs Health Care System, University of South Carolina, Columbia, SC USA; 3 Bioengineering Department, Clemson University, Clemson, SC USA; 4 301 Rhodes Engineering Research, 29634 Clemson, SC USA

**Keywords:** Interpretable machine learning, Heart failure, Biomarkers, Cardiac resynchronization therapy, Personalized medicine

## Abstract

**Background:**

Cardiac Resynchronization Therapy (CRT) is a widely used, device-based therapy for patients with left ventricle (LV) failure. Unfortunately, many patients do not benefit from CRT, so there is potential value in identifying this group of non-responders before CRT implementation. Past studies suggest that predicting CRT response will require diverse variables, including demographic, biomarker, and LV function data. Accordingly, the objective of this study was to integrate diverse variable types into a machine learning algorithm for predicting individual patient responses to CRT.

**Methods:**

We built an ensemble classification algorithm using previously acquired data from the SMART-AV CRT clinical trial (n = 794 patients). We used five-fold stratified cross-validation on 80% of the patients (n = 635) to train the model with variables collected at 0 months (before initiating CRT), and the remaining 20% of the patients (n = 159) were used as a hold-out test set for model validation. To improve model interpretability, we quantified feature importance values using SHapley Additive exPlanations (SHAP) analysis and used Local Interpretable Model-agnostic Explanations (LIME) to explain patient-specific predictions.

**Results:**

Our classification algorithm incorporated 26 patient demographic and medical history variables, 12 biomarker variables, and 18 LV functional variables, which yielded correct prediction of CRT response in 71% of patients. Additional patient stratification to identify the subgroups with the highest or lowest likelihood of response showed 96% accuracy with 22 correct predictions out of 23 patients in the highest and lowest responder groups.

**Conclusion:**

Computationally integrating general patient characteristics, comorbidities, therapy history, circulating biomarkers, and LV function data available before CRT intervention can improve the prediction of individual patient responses.

**Supplementary information:**

The online version contains supplementary material available at 10.1186/s12911-022-02015-0.

## Background

Cardiac resynchronization therapy (CRT) is the preferred treatment method for patients with ventricular dyssynchrony accompanied by reduced ejection fraction and bundle branch block [1]. CRT reduces the risk of sudden heart failure due to the weakening of the heart muscle and can help alleviate disease symptoms for an improved quality of life [2]. The 2008 American Heart Association / American College of Cardiology and 2007 European Society of Cardiology guidelines recommend the following criteria for selecting patients for CRT: patients with sinus rhythm, left ventricular ejection fraction ≤ 35%, QRS > 120ms, NYHA class III/IV [3]. Unfortunately, roughly one-third of CRT recipients do not respond favorably to the treatment [4]. Given its expense and surgical risks, the ability to accurately predict individual patient benefits from this treatment could hold great clinical value [5].

The field of biomedical science has seen a surge in predictive models for disease prognosis and treatment outcomes using various machine learning approaches for detecting subtle patterns in underlying datasets [6, 7]. Recent studies have tested the utility of advanced machine learning algorithms for predicting the response to CRT using various patient data including electronic health records, clinical imaging, electrocardiograms, etc., and some of these studies have reported moderate predictive accuracy [8–14]. However, notably absent in most CRT predictive algorithms is the inclusion of biochemical features. Given the important roles that biochemical markers such as extracellular matrix proteins and inflammatory signals can play in cardiac tissue remodeling, Spinale and colleagues recently showed that the circulating levels of several serum protein biomarkers can hold exciting predictive capability for CRT response [15]. Notably, elevated levels of the soluble suppressor of tumorigenicity-2 (sST-2), soluble tumor necrosis factor receptor-II (sTNFr-II), matrix metalloproteinases-2 (MMP-2), and C-reactive protein (CRP) indicated a reduced likelihood of benefit across ~ 800 patients from the SMART-AV CRT trial.

Past efforts to predict individual patient responses to CRT using machine learning algorithms have largely been limited in two ways. First, most studies have used only a single type of data to make predictions (e.g., electrocardiograms or biochemical markers - not both). Second, most studies have used sophisticated ‘black-box’ algorithms with a limited ability for interpretation or explanation, which can potentially hinder their adoption and utility in clinical practice. One approach for improving interpretability is using simpler models like regression approaches, but an increase in recent explainability approaches is helping to make any model interpretable without penalizing prediction accuracy [16–18].

In this study, our objective was to computationally predict individual patient responses to CRT using a combination of demographics, physical characteristics, comorbidities, medication history, circulating biomarker levels, and echo-based LV assessment. Building upon the previous work of Spinale et al., we combined their biomarker-based metric with various features from the SMART- AV clinical patient data [15, 19]. We assessed the performance of our resulting ensemble machine learning classification model using receiver-operating curve analysis for a hold-out patient dataset and comparisons of 6-month cardiac measures between model-predicted responder and non-responder groups. We also performed SHapley Additive exPlanations (SHAP) analysis to help interpret the global importance of all features included in the model.

## Methods

### Study population and data preparation

The data source for our model training and testing was the SMART-AV trial published previously [19]. In that study, 794 patients with NYHA class II and IV, LVEF ≤ 35%, and QRS duration ≥ 120 milliseconds were randomly assigned to different defibrillation protocols and evaluated at 0, 3, and 6 months with echocardiography and serum biomarker panels. The complete list of recorded features is organized in Table 1 with summary statistics in Table 2. A positive CRT response was defined as a decrease in ESV of at least 15 mL between 0 and 6 months post-surgery, and the patient cohort held a nearly equal split of responders (n = 398) and non-responders (n = 396).

**Table 1:** Variables acquired from the SMART-AV clinical trial

Domain	Individual Feature
General Characteristics (10)	Sex, Age, Height, Weight, Systolic Blood Pressure (BPsys), Diastolic Blood Pressure (BPdia), Heart Rate at Rest (HRrest), QOL Score, 6-Minute Walk Distance, Center size
Comorbidities (10)	Atrial Fibrillation (Afib), Paroxysmal Atrial Fibrillation (PAF), Atrial Flutter, Renal Disease, Chronic Obstructive Pulmonary Disease (COPD), Premature Ventricular Contractions (PVC), Atrial Tachycardia Paroxysmal Supraventricular Tachycardia (AT-PSVT), History of Left Bundle Branch Block (LBBB), History of Right Bundle Branch Block (RBBB), Ischemic Cardiomyopathy
Surgical History (3)	Sinoatrial (SA) Node Surgery, Coronary Artery Bypass Graft (CABG), Pre-Cutaneous Coronary Intervention (PCI)
Medications (3)	Diuretics, Ace inhibitors or ARBs (ACE-ARB), Digoxin
Echo-based Assessment (6)	Left Ventricular End Diastolic Volume (LVEDV), Left Ventricular End Systolic Volume (LVESV), Left Ventricular Ejection Fraction (LVEF), Stroke Volume (SV), EDV/ESV, 1-Dimensional Stretch (cube root of EDV/ESV)
ECG (12)	AV Interval without Atrial Pacing, PR Interval without Atrial Pacing, QRS Width, VT None, VT Nonsustained, VT Supraventricular Tachycardia (VT-SVT), Sick Sinus, Paced AV Delay, Echo Optimized AV Delay, Iterative AV Delay, Fixed AV Delay, Sensed AV Delay
Circulating Biomarkers (12)	**Matrix Metalloproteinase 2 (MMP-2)**, Matrix Metalloproteinase 9 (MMP9), **Soluble** **Suppression of Tumorigenicity 2 (sST-2), C-Reactive Protein (CRP)**, N-terminal pro B-type Natriuretic Peptide (NT- proBNP), Tissue Inhibitors of Metalloproteinase 1 (TIMP1), Tissue Inhibitors of Metalloproteinase 2 (TIMP2), Tissue Inhibitors of Metalloproteinase 4 (TIMP4), Soluble Glycoprotein 130 (sGP130), Soluble Interleukin 2 Receptor Alpha (sIL2Ra), **Tumor Necrosis Factor Receptor II (sTNFR-II)**, Interferon Gamma (IFNg)

**Table 2:** Baseline characteristics of CRT Responders and Non-Responders

Feature Name	All (n = 794)	CRT Non-Responder (n = 396)	CRT Responder (n = 398)
**Continuous Variables, unit**			
Age, year	65.8 ± 10.8	65.6 ± 10.7	66.1 ± 10.9
Height, cm	171.4 ± 10.4	171.6 ± 10.0	171.3 ± 10.7
Weight, kg	87.4 ± 20.8	88.2 ± 20.6	86.6 ± 20.9
BPSys, mm Hg	123.9 ± 20.3	123.2 ± 19.4	124.6 ± 21.0
BPDia, mm Hg	71.4 ± 13.4	71.3 ± 13.5	71.5 ± 13.3
HRrest, bpm	71.1 ± 12.3	70.9 ± 12.7	71.3 ± 11.0
QOL Score	46.6 ± 24.9	50.0 ± 25.8	43.3 ± 23.5
6 MW, m	273.4 ± 124.6	262.3 ± 133.1	284.5 ± 114.6
LVEDV, mL	176.7 ± 72.1	162.9 ± 66.9	190.5 ± 74.5
LVESV, mL	131.6 ± 65.6	118.0 ± 60.0	145.1 ± 68.2
LVEF, %	27.7 ± 8.8	29.7 ± 9.3	25.6 ± 7.8
SV, mL	45.1 ± 14.1	44.9 ± 14.6	45.3 ± 13.6
EDV/ESV Ratio	1.4 ± 0.2	1.5 ± 0.2	1.4 ± 0.2
1D Stretch	1.1 ± 0.0	1.1 ± 0.1	1.1 ± 0.0
AV Interval (Without Atrial Pacing), ms	252.5 ± 69.1	252.7 ± 70.6	252.2 ± 67.6
PR Interval (Without Atrial Pacing), ms	197.2 ± 49.8	200.2 ± 51.3	194.2 ± 48.3
QRS Width (Without Atrial Pacing), ms	153.6 ± 27.3	150.6 ± 26.7	156.7 ± 27.6
Iterative AV Delay (Recommended), ms	127.7 ± 38.2	131 ± 36.3	123.4 ± 39.6
Paced AV Delay (Recommended), ms	174.9 ± 39	181.2 ± 41.2	168.6 ± 37.7
Sensed AV Delay (Recommended), ms	127.1 ± 37.3	132.4 ± 39	121.8 ± 34.9
Biomarker CRT Score (0,1,2,3,4)	1.7 ± 1.2	2 ± 1.1	1.4 ± 1.1
**Binary Categorical Variables, n (%)**			
Sex (Male)	565 (67.4%)	281 (71%)	254 (63.8%)
Atrial Fibrillation (Afib)	99 (12.5%)	57 (14.4%)	42 (10.6%)
PAF	97 (12.2%)	56 (14.1%)	41 (10.3%)
Atrial Flutter	10 (1.3%)	9 (2.3%)	1 (0.3%)
Renal Disease	117 (14.7%)	63 (15.9%)	54 (13.6%)
COPD	115 (14.5%)	64 (16.2%))	51 (12.8%)
PVC	13 (1.6%)	8 (19.7%)	5 (1.3%)
AT-PSVT	12 (1.5%)	8 (2%)	4 (1%)
LBBB	611 (77%)	272 (68.7%)	339 (85.2%)
RBBB	103 (13%)	78 (19.7%)	25 (6.3%)
Ischemic Cardiomyopathy	445 (56%)	271 (68.4%)	174 (43.7%)
SA Surgery	93 (11.7%)	58 (14.6%)	35 (8.8%)
CABG	257 (32.4%)	163 (41.2%)	94 (23.6%)
PCI	248 (31.2%)	155 (39.1%)	93 (23.4%)
Diuretic	637 (80.2%)	316 (79.8%)	321 (80.7%)
ACE-ARB	674 (84.9%)	325 (82.1%)	349 (87.7%)
Digoxin	176 (22.2%)	96 (24.2%)	80 (20.1%)
Small Centersize	176 (22.2%)	86 (21.7%	90 (22.6%)
Sick Sinus	53 (6.7%)	24 (6.1%)	29 (7.3%)
VT None	654 (82.4%)	311 (78.5%)	343 (86.2%)
VT Non-Sustained	95 (12%)	51 (12.9%)	44 (11.1%)
VT SVT	3 (0.4%)	2 (0.5%)	1 (0.3%)
Echo Optimized AV Delay Group	261 (32.9%)	128 (32.3%)	133 (33.4%)
Fixed AV Delay Group	262 (33.0%)	139 (35.1%)	123 (30.9%)

Missing data were imputed using two different methods for our study. Surgical intervention features, PCI and CABG, were imputed to match the most frequent value for each of those features. Categorical data were transformed using one-hot encoding. Non- categorical data and continuous data were imputed using the mean value for each respective variable, followed by the scaling using the RobustScaler method [20]. The patients were split into training and testing datasets, with 80% in the training dataset (n = 635) and 20% in the testing dataset (n = 159). The testing set was completely excluded from model training and feature selection.

### Machine learning model development

**Fig. 1:**
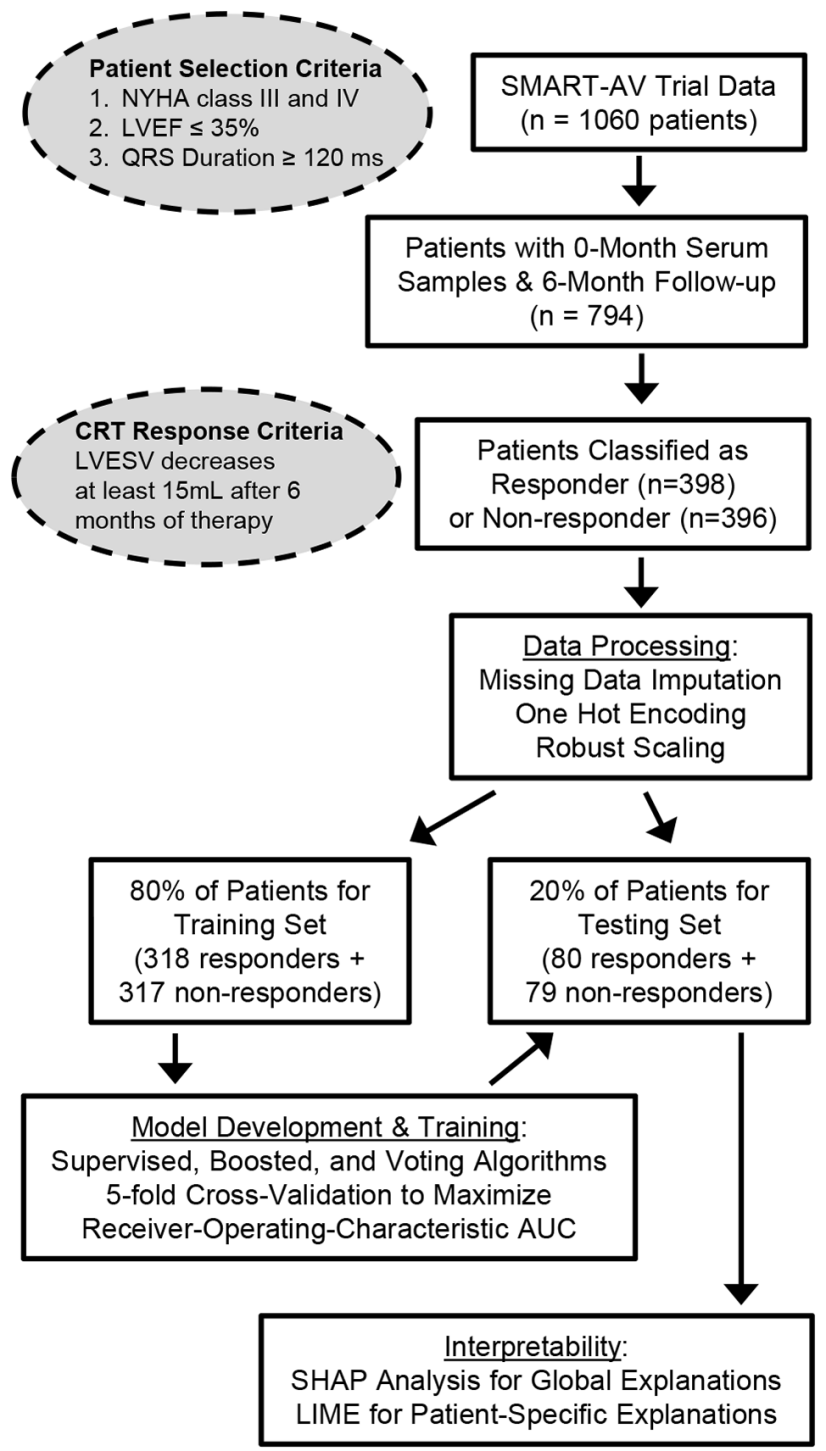
General workflow of algorithm development and testing Patients were previously enrolled in the SMART-AV clinical trial based upon their New York Heart Association (NYHA) heart failure designation, left ventricular ejection fraction (LVEF), and duration of the Q-R-S wave from electrocardiography, and patients were then classified as responders or non-responders based upon their change in left ventricular end-systolic volume (LVESV) after six months of therapy [19]. We first processed the dataset by imputing missing values, numerically encoding categorical variables, and data scaling, and then we separated patients into the training set (for model parameter fitting) and testing set (for model performance testing). Lastly, we used SHapley Additive exPlanations (SHAP) analysis and Local Interpretable Model-agnostic Explanations (LIME) to improve model interpretation through feature explanation

The complete workflow of our model development, testing, and interpretation framework is presented in Fig. 1. Using Python 3.6.4 and scikit-learn 0.23.2, we tested a wide variety of supervised classification machine learning algorithms, including K-Nearest Neighbors, Support Vector Classifier, Decision Tree Classifier, Random Forest, Adaptive Boosting, Gradient Boosted Classifier, Gaussian Naive Bayes classifier, Linear Discriminant Analysis, XGBoost, Catboost, logistic regression, and Multi-Layer Perceptron Neural Network [20]. We also tested Stacked and Voting ensembles that combined these other approaches [21–23]. This list of algorithms includes well-established methods for binary classification tasks where parameters are fit to the underlying classifier structures in order to optimize predictive performance. In general, “ensemble” algorithms seek to improve classification performance by combining several individual algorithms, thereby leveraging the different strengths of each underlying algorithm. “Boosting” approaches, generally, are techniques to improve relatively weak classifiers by iteratively re-weighting data, thereby enabling the algorithm to adapt over successive iterations of model training.

Each model was tuned using a cross-validated grid search across hyperparameters with parameters selected to maximize the area under the receiver-operating characteristic curve (AUC) for binary classification of patients in the training set. Notably, the algorithm only used 0-month (pre-surgery) feature data to predict the 6-month post-surgery response vs. non-response outcome. The resulting model parameters and hyperparameters are provided in Supplemental Table 1.

Feature selection was performed using a backward stepwise methodology, eliminating features that did not improve the model training score. A guiding hypothesis for this work was that combining the previously identified serum biomarkers with demographic and echo-based features would improve predictive capability. To evaluate this hypothesis, we trained and tested our ensembled model using three different sets of features, including all features listed in Table 1 plus (1) no biomarker values, (2) all 12 biomarker values, or (3) a biomarker score based on previous analysis by Spinale et al. [15]. The biomarker score for each patient is calculated by counting how many of the four critical biomarker analytes exceed a risk threshold (MMP-2 ≥ 982,000 pg/mL, sST-2 ≥ 23,721 pg/mL, CRP ≥ 7381 ng/mL, sTNFR-II ≥ 7,090 pg/mL).

### Model interpretation

Model performance was evaluated using 5-fold cross-validation within the training dataset, and the final model was selected based on the highest mean AUC. After model selection using the training set, the final model performance was validated using the holdout validation set. Interpretation of model output results is difficult with ensemble models due to the inherent complexity of layering multiple algorithms to select a prediction. To help interpret global feature importance, we performed a SHapley Additive exPlanations (SHAP) analysis using the Python SHAP library 0.37.0 KernelExplainer and KernelSHAP using all samples as input for SHAP value calculation [24]. With ensemble models, feature importance and predictions become very personalized to the individual sample making it difficult to understand a local prediction using only global feature importance. To provide a more personalized explanation of an individual prediction, local interpretation is more accurate. We also picked two examples of CRT recipients to demonstrate how the model behaves locally for responders and non-responders using Local Interpretable Model-agnostic Explanations (LIME) [25].

## Results

### Model predictive performance

**Fig. 2:**
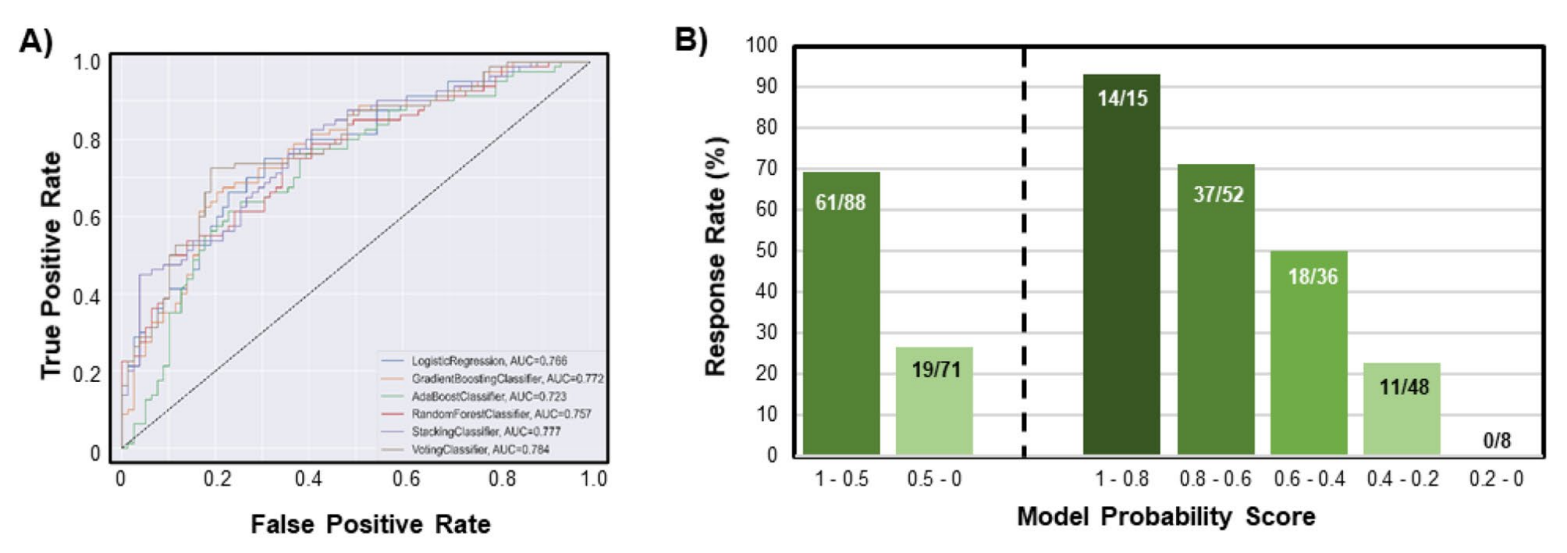
Overall performance of the machine learning model The Receiver-Operating Characteristic curve for the supervised, binary classification ensemble model demonstrates high predictive capability with an area-under-the-curve of 0.784 for the majority voting classifier. (B) Model-predicted responders exhibited a 69% response rate (61/88), while model-predicted non-responders exhibited only a 27% response rate (19/71). Further stratification based on the model-predicted responses probability score demonstrated a greater predictive accuracy

Across all the algorithms tested, a majority-voting ensemble classification model demonstrated the best performance. The ensemble consisted of nine equally weighted models, each voting with their respective probability of surgical success: a Linear Discriminant Analysis classifier, a Catboost Classifier, a Gradient Boosted classifier, a Random Forest classifier, an XGBoost classifier, a Support Vector Classifier, a 3-layer Multi-level Perceptron Neural Network, a Logistic Regression Classifier, and an Adaboost classifier. Without using biomarker data, our algorithm approach demonstrated modest predictive performance with an AUC of 0.63 in the training patient set (Table 3). The addition of biomarker data substantially improved model performance with an AUC reaching 0.75 in the training patient set using all 12 biomarkers or the simplified biomarker composite score (Table 3). Using the biomarker score with a voting classifier reached the highest AUC in both the training and test patient set, so we proceeded with this model for the remaining analyses (Table 4; Fig. 2 A).

**Table 3:** Area-Under-the-Curves (AUC) for the ML models with or without the biomarker data

Feature Set	Biomarker Feature Used	Train AUC (n = 635)	Test AUC (n = 159)
No Biomarkers	None	0.63	0.74
All Biomarkers	MMP-2, MMP9, sST-2, CRP, NT- proBNP, TIMP1, TIMP2, TIMP4, sGP130, sIL2Ra, sTNFR-II, IFNg	0.75	0.77
Biomarker Score (0,1,2,3,4)	MMP-2 (≥ 982,000 pg/mL), sST-2(≥ 23,721 pg/mL), CRP (≥ 7381 ng/mL), sTNFR-II (≥ 7,090 pg/mL)	0.75	0.78

**Table 4:** Comparison of the performance of the top 6 models in our study using Biomarker Scoring

Model Name	Accuracy Train	Accuracy Test	Recall Train	Recall Test	ROC-AUC Test	F1 Test	MCC Test
Voting Classifier	**1.000**	**0.730**	0.997	0.713	**0.784**	0.726	**0.460**
Stacking Classifier	0.855	0.723	0.915	**0.800**	0.772	**0.744**	0.451
Gradient Boosting Classifier	**1.000**	**0.730**	**1.000**	0.625	0.775	0.699	0.470
Logistic Regression	0.706	0.692	0.733	0.763	0.766	0.713	0.387
Random Forest Classifier	0.935	0.679	0.940	0.750	0.757	0.702	0.361
Adaptive Boosting Classifier	0.751	0.667	0.802	0.775	0.723	0.701	0.340

Our binary classification model correctly predicted 71% of patient responses in the test set, with 61/88 classified responders and 52/71 classified non-responders matching the trial result (Fig. 2B). In other words, the prediction yielded 61 true positives, 52 true negatives, 27 false positives, and 19 false negatives. To analyze more detailed patient stratifications, we separated patients into five groups according to the model-predicted probability of response (i.e., probability bins = 1-0.8, 0.8 − 0.6, 0.6 − 0.4, 0.4 − 0.2, or 0.2-0). Across the stratified patients, the model correctly identified 96% of patients in the highest and lowest response groups, with 14/15 patient responders in the high probability score group and 8/8 non-responders in the low probability score group (Fig. 2B).

**Fig. 3:**
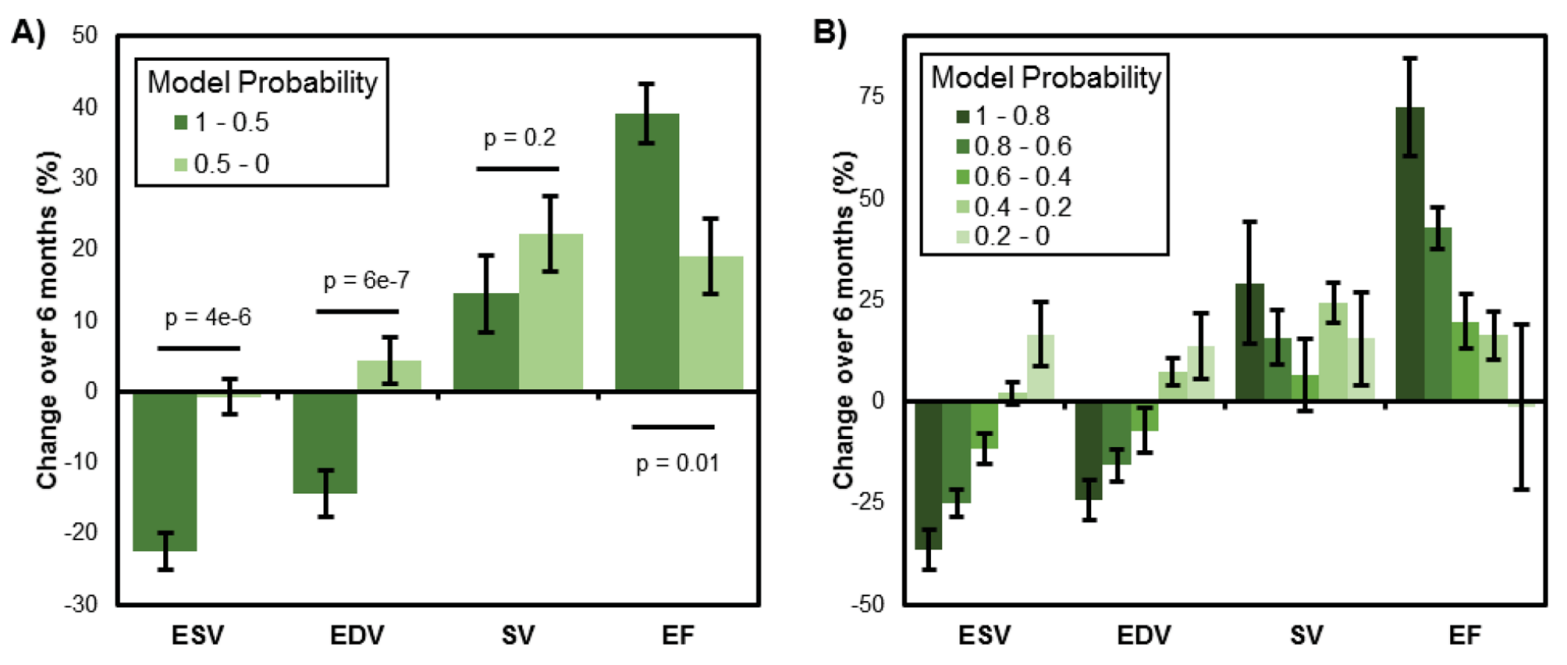
Cardiac remodeling across patient stratifications Model-predicted responders showed statistically significant differences in left ventricle remodeling metrics compared to the model-predicted non-responders. (A) Binary classification identified a responder group with substantially greater improvements in ESV, EDV, and EF from 0–6 months after CRT intervention. (B) More detailed patient stratification further amplified the remodeling differences across groups

In addition to response rate (which is judged by a strict over/under -15mL threshold for ESV change over six months), we also explored quantitative changes in left ventricle remodeling metrics across the model classification groups (Fig. 3). Over six months after the procedure, patients predicted by the model as responders showed significant reductions in both ESV and EDV, while patients classified as non-responders showed no change in ESV and a slight increase in EDV over six months. Both responders and non-responders showed increased stroke volumes and ejection fractions, but the model-predicted responders showed a statistically more significant improvement in ejection fraction (~ 40% compared to ~ 20%). These discrepancies between groups were amplified further across the 5-group patient stratification using the model probability score (Fig. 3B). In the most extreme case, the high response probability group exhibited almost a 75% improvement in ejection fraction, while the low response probability group exhibited no change in ejection fraction over the 6 months after surgery.

### Model interpretability

**Fig. 4:**
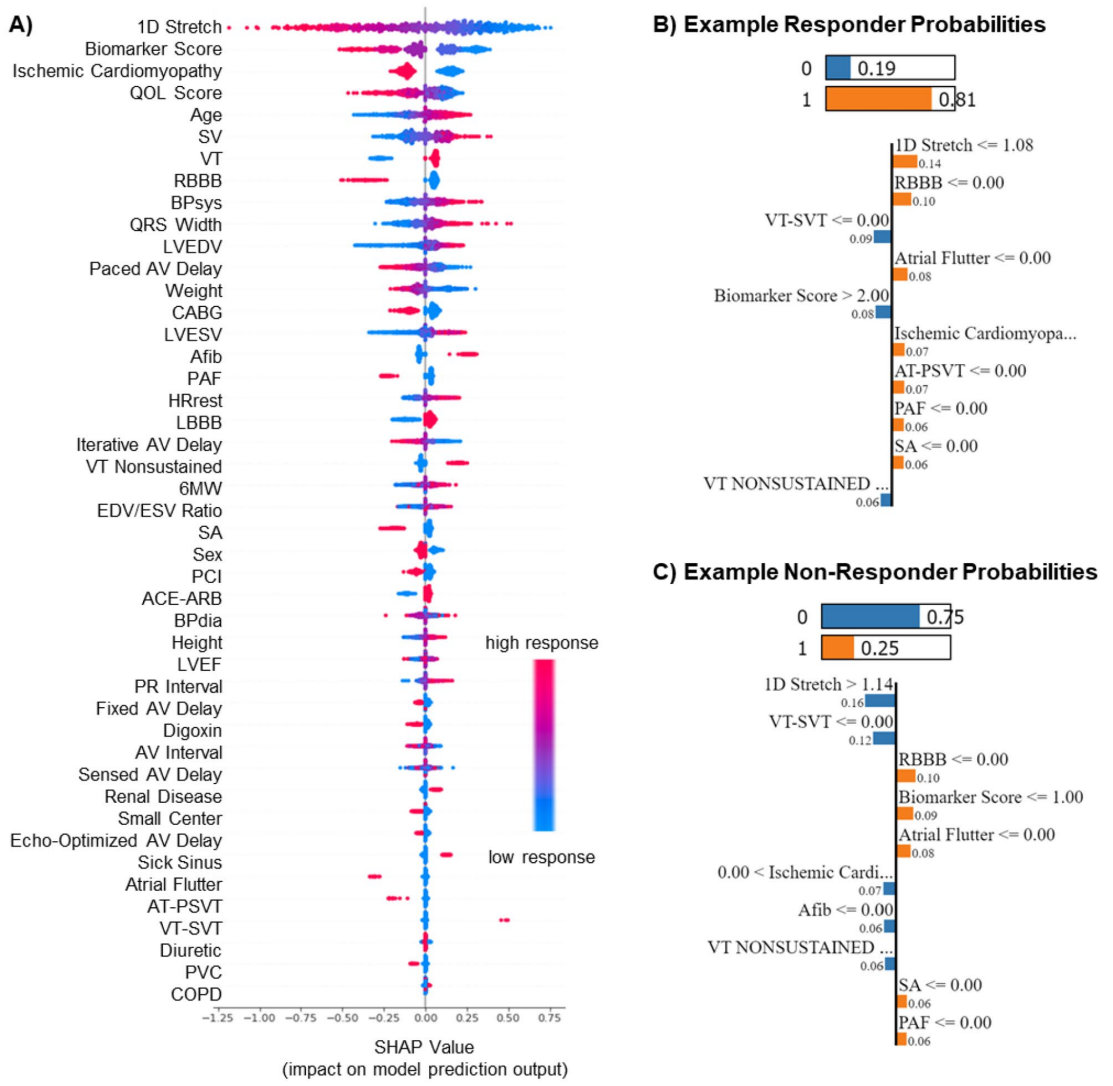
Global and local interpretations of model predictions (A) SHAP plot shows the feature importance in our model. 1D stretch, biomarker score, ischemic cardiomyopathy, QOL score, and age were indicated as the top 5 most important features for determining patient response probability. The scatter width and separation indicate the feature importance, and the color indicates which direction of that feature value is predictive of high vs. low patient response. (B) LIME plot shows the most significant contributing features for an example responder wherein a 1D Stretch of ≤ 1.08 along with a lack of RBBB, atrial flutter, ischemic cardiomyopathy, AT_PSVT, PAF, and SA surgery increased the probability of responding favorably to CRT treatment. (C) LIME plot shows the most significant contributing features for an example non-responder wherein a 1D Stretch of > 1.14 along with a lack of VT-SVT, Afib, and nonsustained VT increased the probability of not responding to treatment. On the other hand, a history of ischemic cardiomyopathy also affected the predicted non-response to CRT.

To improve the interpretability of our ensemble classification algorithm, we performed a SHAP analysis and corresponding visualization of feature importance (Fig. 4 A). Briefly, this technique calculates a collective, global average of how much each feature value contributed to each patient’s classification to indicate both the magnitude and direction that each feature contributes to the overall probability of falling on either side of the binary classifier (i.e., responders vs. non-responders). SHAP analysis indicated that lower 1D stretch, lower biomarker score, absence of ischemic cardiomyopathy, lower QOL score, and higher age were strong global contributors within the algorithm for identifying responders.

To demonstrate feature importance in a local, patient-specific visualization, we use LIME for an example responder and non-responder (Fig. 3B). For the responder patient example with a higher probability of responding to CRT (0.81), 1D stretch of less than or equal to 1.08 and no history of RBBB, Atrial Flutter, Ischemic Cardiomyopathy, AT-PSVT, PAF, and SA are helping to move the patient to the response regimen. But no history of VT-SVT, non-sustained VT, and Biomarker Score > 2 contribute to non-responsiveness for this patient. For the non-responder patient example with a higher probability of not responding to CRT (0.75), 1D stretch of greater than 1.14, a history of Ischemic Cardiomyopathy, and no history of VT-SVT, Afib, and Nonsustained VT are helping to move the patient to the non-response group. But no history of RBBB, Atrial Flutter, SA surgery, PAF, and Biomarker Score of zero is responsible for this patient’s small probability of response to CRT.

## Discussion

While CRT offers significant clinical benefits for many heart failure patients, a large proportion of the population does not respond positively to treatment [4]. This high patient-to-patient variability presents a need for predictive methods to help identify which patients will or will not benefit from CRT based on information obtained before the procedure. We hypothesized that integrating multiple data sources and including biochemical levels from serum panels would significantly improve the predictive ability of machine learning algorithms.

Using previously obtained patient data in the SMART-AV trial, we built a novel algorithm that integrates demographic data, physical characteristics, medical history, circulating biomarker levels, and echocardiography data to improve the prediction of CRT response before surgical intervention. In a previous study, Spinale and colleagues showed significant predictive power for identifying CRT response using pre-surgical levels of specific serum protein biomarkers (sST-2, sTNFr-II, MMP-2, and CRP) [15]. Given the important roles of inflammation and extracellular matrix turnover for regulating cardiac remodeling related to CRT, it should be no surprise that circulating proteins are associated with CRT response either as upstream regulators or downstream correlates. We combined the Spinale et al. patient biomarker score with 40 other input features spanning echo-based LV metrics, medical history, demographic information, and basic clinical assessments. Using these features enabled our ensemble machine learning classifier to correctly identify 71% of patient response outcomes, achieving an AUC of 0.784 – a substantial improvement over the previous study using the biomarker score alone [15].

A major limitation of many machine learning approaches is their ‘black-box’ nature of predictions, or in other words, their un-explainability. Future adoption of artificial intelligence into the clinical decision-making process will undoubtedly be affected by an ability to explain (to some degree at least) why models predict what they predict and to identify the driving variables within the algorithms, especially for high-risk and costly decisions like CRT treatment. To improve interpretability in high risk or costly decisions, a growing emphasis is being put on ‘glass-box’ or ‘white-box’ techniques. We employed SHAP analysis to elucidate the relative contribution of each feature globally to the patient response probability output of our model (Fig. 4). This analysis revealed that important features came from diverse data sources, with the top five features including echo-based data (1D stretch), serum protein data (biomarker score), co-morbidity data (ischemic cardiomyopathy), clinical evaluation data (QOL score), and demographic data (patient age). In addition, LIME revealed features responsible for personalized prediction and showed diverse feature sets responsible for individual response to treatment. Of course, we must emphasize that the power of these features to predict CRT response is indicative of their correlation to cardiac remodeling and not necessarily indicative of their mechanistic causation of cardiac remodeling. Additional notable limitations include a relatively short follow-up time of 6 months and a relatively small patient sample size (compared to thousands of patients’ data used in electronic health record-based algorithms).

Numerous recent studies have applied a wide range of machine learning approaches to predict CRT from diverse datasets [8–14]. All these reports have generally produced AUC values > 0.7 with the best performing algorithms ~ 0.8% (comparable to our 0.784 AUC). The datatypes used for these past reports have varied (electronic health records, clinical imaging, demographic data, electrocardiograms, etc.), and the computational algorithms have spanned a range of simple regression models to more complicated approaches including gradient boosting [8], Naïve-Bayes [9], multiple kernel learning [10], random forest [11], adaptive lasso [12], and support vector machines [13]. In agreement with our results, the most important predictors from past studies have spanned different data types across comorbidity (e.g., ischemic cardiomyopathy and LBBB), electro-mechanical (e.g., systolic blood pressure, QRS width, and wall strains), and demographic data (e.g., age and sex) [8, 10, 12]. This diversity of predictor type further supports our underlying hypothesis that various data sources are not necessarily redundant and can therefore provide additive benefit for identifying CRT response.

Current clinical guidelines define specific eligibility criteria for physicians to base their CRT recommendations [26]. The increasing accuracy of computational predictions suggests that incorporating personalized model-based probabilities could benefit such recommendation criteria. Encouragingly, our patient stratification demonstrated 96% accuracy in the highest and lowest response subgroups with significant differences in volume changes and functional changes over six months post-CRT. Using higher resolution (quintile) binning was motivated by the potential practical utility for a clinician to label patients as very high, high, neutral, low, and very low response categories. Clinician decisions are often more complicated than simply “operate vs. do not operate”, so the intermediate group binning could inform when to take other clinical options (e.g. additional measurements, prolonged observation, etc.). Our algorithm was built and tested using only baseline, pre-CRT measurements, demonstrating that it is feasible for machine learning algorithms to harness a composite set of data from the demographic, functional, and biomarker domains obtained at the time of patient evaluation for CRT and provide predictive value on the ultimate CRT response. As future model developments are likely to further improve prediction accuracy across a broader number of patients, future clinical and ethical discussions will prove vital to appropriately leverage this predictive information into CRT decisions.

## Conclusion

In this study, we have shown that integrating multiple types of data including demographics, circulating biomarkers, and echo-based structure features can improve the predictive capability of machine learning algorithms to identify CRT responders and non-responders before intervention. Further, interpretability approaches like SHAP and LIME can help elucidate specific contributions of each feature’s role in determining the predicted responses across a cohort and patient-specific level.

## Electronic supplementary material

Below is the link to the electronic supplementary material.

Supplementary Material 1

## Data Availability

Computational codes used in this study are available on GitHub: https://github.com/SysMechBioLab/CRT_IML.

## Abbreviations

BPsys: Systolic blood pressure
BPdia: Diastolic Blood Pressure
HRrest: Heart Rate at Rest
QOL: Quality of Life
6MW: 6-Minute Walk Distance
Afib: Atrial Fibrillation
PAF: Paroxysmal Atrial Fibrillation
COPD: Chronic Obstructive Pulmonary Disease
PVC: Premature Ventricular Contractions
AT-PSVT: Atrial Tachycardia-Paroxysmal Supraventricular Tachycardia
LBBB: History of Left Bundle Branch Block
RBBB: History of Right Bundle Branch Block
SA: Sinoatrial Node Surgery
CABG: Coronary Artery Bypass Graft
PCI: Pre-Cutaneous Coronary Intervention (PCI)
LVEDV: Left Ventricular End Diastolic Volume
LVESV: Left Ventricular End Systolic Volume
LVEF: Left Ventricular Ejection Fraction
SV: Stroke Volume
AV: Atrioventricular
PR: P-wave to QRS complex
QRS: Width of Q, R, S wave
VT: Ventricular Tachycardia
