# Supplementary material for: Interpretable machine learning predicts cardiac resynchronization therapy responses from personalized biochemical and biomechanical features
Source: BMC Med Inform Decis Mak. 2022 Oct 31;22:282. doi: 10.1186/s12911-022-02015-0 (PMC9620606; doi:10.1186/s12911-022-02015-0)
Supplement: Supplementary file 1 — Supplementary Material 1 [file 12911_2022_2015_MOESM1_ESM.docx]

Supplemental Table 1: Machine Learning Model Parameters and Hyperparameters

| **Machine Learning Model** | **Parameters and Hyperparameters** |
| --- | --- |
| Random Forest | max_depth=80, max_features=13, min_samples_leaf=5, min_samples_split=12, n_estimators=300, |
| SVC | C=10, gamma='auto', kernel='linear', probability=True |
| KNN | algorithm='ball_tree', leaf_size=15, n_neighbors=15 |
| XGBoost | base_score=0.5, booster='gbtree', colsample_bylevel=1, colsample_bynode=1, colsample_bytree=0.8, eval_metric='auc', gamma=0, gpu_id=-1, importance_type='gain', interaction_constraints='', learning_rate=0.1, max_delta_step=0, max_depth=5, min_child_weight=1, missing=nan, monotone_constraints='()', n_estimators=400, n_jobs=32, num_parallel_tree=1, random_state=0, reg_alpha=1.2, reg_lambda=1.2, scale_pos_weight=1, subsample=0.9, tree_method='exact', use_label_encoder=False, validate_parameters=1, verbosity=None |
| Gradient Boosting | max_depth=80, max_features=3, min_samples_leaf=3, min_samples_split=8, n_estimators=1000 |
| Adaptive Boosting | learning_rate=0.2 |
